# Identification of a circulating carbohydrate antigen as a highly specific and sensitive target for schistosomiasis serology

**DOI:** 10.1128/jcm.01008-24

**Published:** 2025-01-13

**Authors:** Anna O. Kildemoes, Tom Veldhuizen, Stan T. Hilt, Lisette van Lieshout, Taniawati Supali, Maria Yazdanbakhsh, Daniel Camprubí-Ferrer, Jose Muñoz, Joannes Clerinx, Mickey Harvey, Jeroen Codée, Paul L. A. M. Corstjens, Govert J. van Dam, Leo G. Visser, Meta Roestenberg, Angela van Diepen, Cornelis H. Hokke

**Affiliations:** 1 Leiden University Center for Infectious Diseases, Leiden University Medical Center 4501 , Leiden, the Netherlands; 2 Department of Cell and Chemical Biology, Leiden University Medical Center 4501 , Leiden, the Netherlands; 3 Department of Parasitology, Universitas Indonesia 64733 , Jakarta, Indonesia; 4 ISGlobal, Hospital Clínic - Universitat de Barcelona 16724 , Barcelona, Spain; 5 Department of Clinical Sciences, Institute of Tropical Medicine, Antwerp, Belgium; 6 Leiden Institute of Chemistry, Leiden University Medical Center 4501 , Leiden, the Netherlands; Mayo Clinic Minnesota, Rochester, Minnesota, USA

**Keywords:** schistosomiasis, diagnostics, serology, neglected tropical diseases, carbohydrate

## Abstract

The World Health Organization (WHO) 2030 roadmap for schistosomiasis calls for development of highly sensitive and specific diagnostic tools to continue and sustain progress towards elimination. Serological assays are excellent for sensitive detection of primary schistosome infections and for schistosomiasis surveillance in near- and post-elimination settings. To develop accurate assay formats, it is necessary to identify defined antibody targets with low cross-reactivity and potential for standardized production. Here we aim to identify such target(s) with focus on defined schistosome glycan antigens. Target identification was performed by assessing antibody responses in well-characterized cross-sectional and cohort sample sets (*n* = 366 individuals) on tailor-made antigen microarrays. IgM and IgG binding to candidate diagnostic targets was measured for serum/plasma samples from controlled human schistosome infection models, schistosome-infected travelers, soil-transmitted helminth-infected individuals, and non-infected individuals. We found that antibodies to a schistosome gut-associated glycan, the circulating anodic antigen (CAA), identify schistosome infection with high sensitivity (IgM ≥100%, IgG ≥97%) and specificity (IgM ≥93%, IgG ≥97%) in the test samples. Infection dose affected timing of anti-CAA antibody isotype switch. Furthermore, we demonstrate that other non-specific glycan epitopes in crude schistosome cercarial and egg antigen preparations can contribute to generation of false schistosomiasis positives, which is relevant for current serological assays based on these antigen mixtures. In conclusion, CAA is an excellent single glycan antigen target for development of highly sensitive and specific tools for schistosomiasis serology with use cases for travelers and surveillance in near- and post-elimination settings, as well as emerging transmission zones.

**IMPORTANCE:**

The WHO 2030 roadmap deems diagnostics developments for schistosomiasis critically needed. Here we present identification of an antibody target with superior performance compared to traditionally used crude antigens in schistosomiasis serology. Access to unique controlled human infection model samples, traveler samples, and negative controls enabled this discovery, which forms the basis for development of new diagnostic tools urgently needed in travel medicine, surveillance in emerging transmission zones driven by climate change, and in pre- and post-elimination scenarios.

## INTRODUCTION

Schistosomiasis caused by parasitic blood flukes remains a large global public health burden with an estimated cost of 1.9 million disability-adjusted life years and more than 250 million people infected worldwide (1). The current World Health Organization (WHO) 2021–2030 roadmap for schistosomiasis control and elimination advocates for holistic and integrated strategies to tackle the disease burden. Development of diagnostics is identified as a critical need to accelerate progress towards elimination (2). For non-endemic, low-endemic, and near- or post-elimination settings, highly specific diagnostic tools for surveillance are needed. Additionally, strategies combining sensitive first-line screening tests with second-line assays specific for active infections are envisioned to optimize resource use and drug impact (3). Development of a highly specific serological tool for schistosomiasis will fill a gap in the portfolio of currently available diagnostic tools. High sensitivity is an inherent property of robust serological methods, which basically exploit the host-driven signal amplification induced after antigen recognition and subsequent antibody response. However, a well-known limitation of diagnostic serology is the persistence of antibodies in circulation after successful clearance or cure of an infection. As antibody detection cannot distinguish adequately between prior and current infections, it adds the most value in the context of primary exposures such as in non-endemic area travel medicine and surveillance in (re-)emerging transmission risk zones. In endemic regions, serology can be a powerful element in transmission risk mapping as well as near- and post-elimination monitoring, particularly through strategic application in defined target groups such as young children or occupational risk groups (4, 5).

In contemporary schistosomiasis serology, typical antibody-detection platforms are enzyme-linked immunosorbent assays (ELISA), indirect haemagluttinin assays (IHA) and/or immune fluorescence assays (IFAs, commonly using adult worm sections) (6–8). Many of such existing commercial and/or in house serological assays are based on crude soluble egg, cercarial, or worm antigen preparations (9–11). This means that they are based on undefined mixes of antigens, which may contain cross-reactive epitopes and suffer from batch-to-batch variation (12, 13). In schistosome infections, a large proportion of the antibodies elicited recognize glycan elements present in the parasite’s glycoprotein and glycolipid repertoire, as demonstrated both in rodent and primate models, as well as cross-sectional human sample sets (14, 15). High antigenicity is attractive for achieving high sensitivity of a serological assay. However, subsets of glycan elements expressed in schistosomes are shared with other helminths, other invertebrates, foods of plant origin, or the mammalian host (16, 17). Anti-glycan antibodies that recognize glycans shared between schistosomes and other pathogens or commensal organisms are an inherent liability to the specificity of assays based on crude antigen preparations. From a diagnostic perspective, it is therefore essential to identify which glycan targets are highly specific and which are not. Extensive glycomic work has confirmed that, aside from those that also occur in other organisms, schistosomes express several glycan elements which appear unique to the parasite (18). In this study, such glycomics knowledge was combined with anti-glycan antibody data from primate models and human sample sets (14, 15) to design custom microarrays for antibody response evaluation. We exploited access to controlled human schistosome infection (CSI) and primary infection traveler samples to identify a highly specific antibody target. Specifically, the aim of this study is to identify defined glycan antigen(s) which display high specificity (≥95%) and sensitivity (≥95%) for future development of highly accurate antibody-detection tools. Such tools are needed for diagnosis of primary schistosome infections and surveillance in near- and post-elimination settings, as well as emerging transmission zones.

## MATERIALS AND METHODS

Elaborate methodology describing reagents and apparatus can be found in the supplemental material.

### Anonymized sample sets

#### Primary schistosome infection sample sets

(**A1**) Controlled human schistosome infection (CSI1) serum samples from 17 healthy Dutch adult volunteers collected at baseline (bsl) and at weeks 1–8, 10, 12, 14, 16, 18, 20, and 52 post-infection with male-only *Schistosoma (S.) mansoni* cercariae (no egg production). The original study was an open-label dose-escalation clinical safety study carried out at Leiden University Medical Center (LUMC), the Netherlands, where participants were exposed to, respectively, 10 (*n* = 3), 20 (*n* = 11), and 30 (*n* = 3) cercariae at week 0 and treated with praziquantel at week 12 (40 mg/kg body weight) as described in Langenberg et al*.* (19). Exclusion criteria included schistosomiasis history, treatment for schistosomiasis, and positive serology. The trial is registered at clinicaltrials.gov with identifier NCT02755324, and ethical approval was given by the LUMC Institutional Medical Ethical Research Committee (Institutional Review Board P16.111). (**A2**) Sera from the other single-sex schistosome infection open-label, dose-escalation clinical safety study carried out at LUMC, where Dutch volunteers were exposed to female-only *S. mansoni* cercariae (CSI2), were also available. In this trial, participants were exposed to, respectively, 10 (*n* = 3) and 20 (*n* = 7) cercariae at week 0, and they were treated with praziquantel at weeks 8 and 12 (60 mg/kg body weight) as described in Koopman et al. (20). These sera were collected weekly from bsl to week 10 and then at 12, 14, 16, 18, 20, and 52 weeks post-infection. The trial registration number is NCT04269915 at clinicaltrials.gov, and ethical approval was given by LUMC Institutional Medical Ethical Research Committee (Institutional Review Board P20.015).

(**B**) Serum samples originally from the post-travel screening of parasites (PTSP) study published in Soonawala et al*.* (21) carried out in 2007–2009 with recruitment at travel clinics in Leiden and Wageningen, the Netherlands, with specific written opt-in consent for use in other diagnostic research. Samples are from individuals traveling for a minimum of 1 month on the African continent and were obtained pre-travel and 12 weeks after return to the Netherlands (*n* = 131 pre-travel, *n* = 113 paired pre-travel–post-travel). The samples were long-term stored at −80°C and freeze-thawed only a limited number of times. The original study encompassed nine schistosome infection cases diagnosed post-travel by seroconversion (IFA based on adult worm antigen sections [IgM] [22] and/or soluble egg antigen [SEA]-ELISA [IgG] [23]) all with histories of swimming in Lake Malawi and/or Lake Victoria. Two of the nine seropositive individuals were also *Schistosoma* spp. DNA PCR positive.

(**C**) *S. mansoni*-clade primary infection time-course serum sample set from six 18-year-old male Spanish adults with no previous medical history of schistosomiasis, who were exposed when swimming in Chicamba Lake, Mozambique, on 14 July 2019. All individuals were egg negative and presented with acute schistosomiasis symptoms as described in Camprubí-Ferrer et al. (24). The participants were part of a larger cohort study on febrile travelers and approval was obtained through the Hospital Clinic of Barcelona Institutional Review Board and Ethics Committee (HCB/2017/0612).

(**D**) Serum samples from *S. haematobium × S*. *mattheei* primary exposure/infection in a cluster of Belgian tourists visiting South Africa exposed on two different occasions through swimming and rafting in uMhkunyane river (*n* = 34, consisting of 8 families, end of 2016/early 2017). All individuals were egg negative and 32 of 34 presented with acute schistosomiasis symptoms as published in Cnops et al. and Hoekstra et al*.* (25, 26). Informed consent including storage and use for future schistosomiasis diagnostic assays was given by all participants at the Institute of Tropical Medicine (ITM), Antwerpen, Belgium.

#### Schistosome infection-negative sample sets

(**E**) Soil-transmitted helminth (STH) infection-positive (*Ascaris lumbricoides*, *Trichuris trichiura*, *Necator americanus*, *Ancylostoma duodenale*, *Strongyloides stercoralis*) plasma samples from the Immunospin study as published in Wiria et al. with participants from Pulau Flores, Indonesia (*n* = 97 from 2009 sample collection) (27). The STH status was determined by RT-PCR for *A. lumbricoides*, *N. americanus*, *A. duodenale*, and *S. stercoralis* and by copro-microscopy performed after formol-ether acetate concentration for *A. lumbricoides*, *T. trichiura*, and the hookworms. Lastly the Harada-Mori method was applied for *S. stercoralis* prior to microscopy in the original study. This island is not endemic for schistosomiasis and the likelihood of participants having a history of schistosome exposure is negligible. The clinical trial reference number is ISRCTN83830814, with ethical clearance through Ethical Committee of the Medical Faculty, University of Indonesia (194/PT02.FK/Etik/2006), and filed by the Committee of Medical Ethics of the Leiden University Medical Center.

(**F**) *Strongyloides stercoralis* (*S. ster.*) infection-positive sera (*n* = 25) as diagnosed by ELISA and/or PCR were retrieved from a biobank based on the laboratory information management system of the clinical microbiology department of the LUMC. These 25 samples were handed to the research team in a fully anonymized way. No schistosomiasis diagnostics was conducted, and no further clinical information was given. Samples were included only if the patient had not specifically indicated that sample material could not be used for other purposes than diagnosis of disease, as regulated by law and stated in (28). Ethical approval was given by the LUMC Institutional Medical Ethical Research Committee (Institutional Review Board B21.047).

(**G**) Sera (NLD donor) were obtained via the Dutch blood donor bank, Sanquin, from healthy blood donors (*n* = 56).

### Construction and incubation of microarrays

Custom microarrays consisting of synthetic glycan elements, keyhole limpet hemocyanin, native schistosome circulating cathodic antigen (CCA) and circulating anodic antigen (CAA), negative controls, and crude schistosome cercarial and egg antigen preparations with and without NaIO_4_ treatment were prepared in 10% dimethyl sulfoxide print-buffer in plates. Nanoliters of each array antigen were then printed onto epoxilane-coated glass slides with a MicroGrid robot in a triplicate/array (15). The full target list can be found in Table S1. For incubation with serum/plasma samples, the glass slides were first fitted with silicone gaskets, reconstituted in phosphate-buffered saline (PBS), and blocked for ≥1 h in PBS with 2% bovine serum albumin and 50 mM ethanolamine. Each array was subsequently washed in PBS-Tween (0.05%) followed by PBS, and the sample in buffer/array was added for a 1 h incubation. The slides were washed and incubated with the relevant anti-human (IgM and IgG [subclass]) antibodies with fluorophores for 30 min. Finally, slides were washed as above with an additional wash in milli-Q before they were spun dry and scanned (Agilent Scan Control).

### Schistosomulae culture and measurement of CAA and CCA

*S. mansoni* cercariae were transformed by heat shock. Schistosomulae and the released tails were separated on an orbital shaker and transferred to a tube with antibiotics and antimycotic (1:100) in Dulbecco’s phosphate-buffered saline (DPBS) for 20 min incubation at 37°C. Schistosomulae were sedimented, and the media was changed to 10 mL media with 0.02% red blood cells for plating into a 48-well plate (200 µL parasites plus 800 µL media/well). Single schistosomulae were collected at baseline and at day 8. For days 1–7, five full replicate wells and a control well were collected. Parasites were counted in 100 µL/well at ×2 magnification. The remaining material was split into supernatant and parasite material by centrifugation at 2,000 × *g*. Parasite pellets were washed twice in DPBS and subsequently freeze-dried, re-suspended in DPBS and sonified. CAA and CCA were measured in supernatant (antigens regurgitated into media by parasites) and in the schistosomulae parasite pellets (antigen content in the parasite gut) by upconverting phosphor lateral flow (UCP-LF) assay using adapted urine to PBS assay protocols.

### Data processing and analyses

Microarray scan images were obtained through Agilent Feature Extraction v.10.7.3.1 and imported into GenePixPro v.7. Spot morphology quality was inspected, and median fluorescence intensity (MFI) values were obtained per nanodot with the average of each triplicate MFI target used for analyses. Further data processing was done in Microsoft Excel and graphs were generated using GraphPad Prism v.9.3.1. Variation is shown as standard deviations, with horizontal bars indicating median values. Response profiles and magnitude of response to single targets can be compared between samples, but different targets within arrays are not directly quantitively comparable due to differences in printing characteristics determining spot density and epitope accessibility. An arbitrary theoretical cut-off value of MFI = 10,000 was chosen to enable comparative basis for specificity and sensitivity calculations for different antigen(s). Area under the curve (AUC) based on mean MFI with 95% confidence intervals (CI) are given for comparison of isotype switch groupings. Wilcoxon matched-pairs signed-rank test was used to compare mean values over time between early and later responders. Welch’s unequal variances *t*-test was used to compare mean CAA/CCA content in individually collected schistosomulae. Comparison of CAA/CCA content and excretion in the multi-well culture experiment was done using Brown-Forsythe and Welch’s analysis of variance (ANOVA) with Dunnett’s T3 multiple comparisons test (GraphPad Prism).

## RESULTS

**Fig 1:**
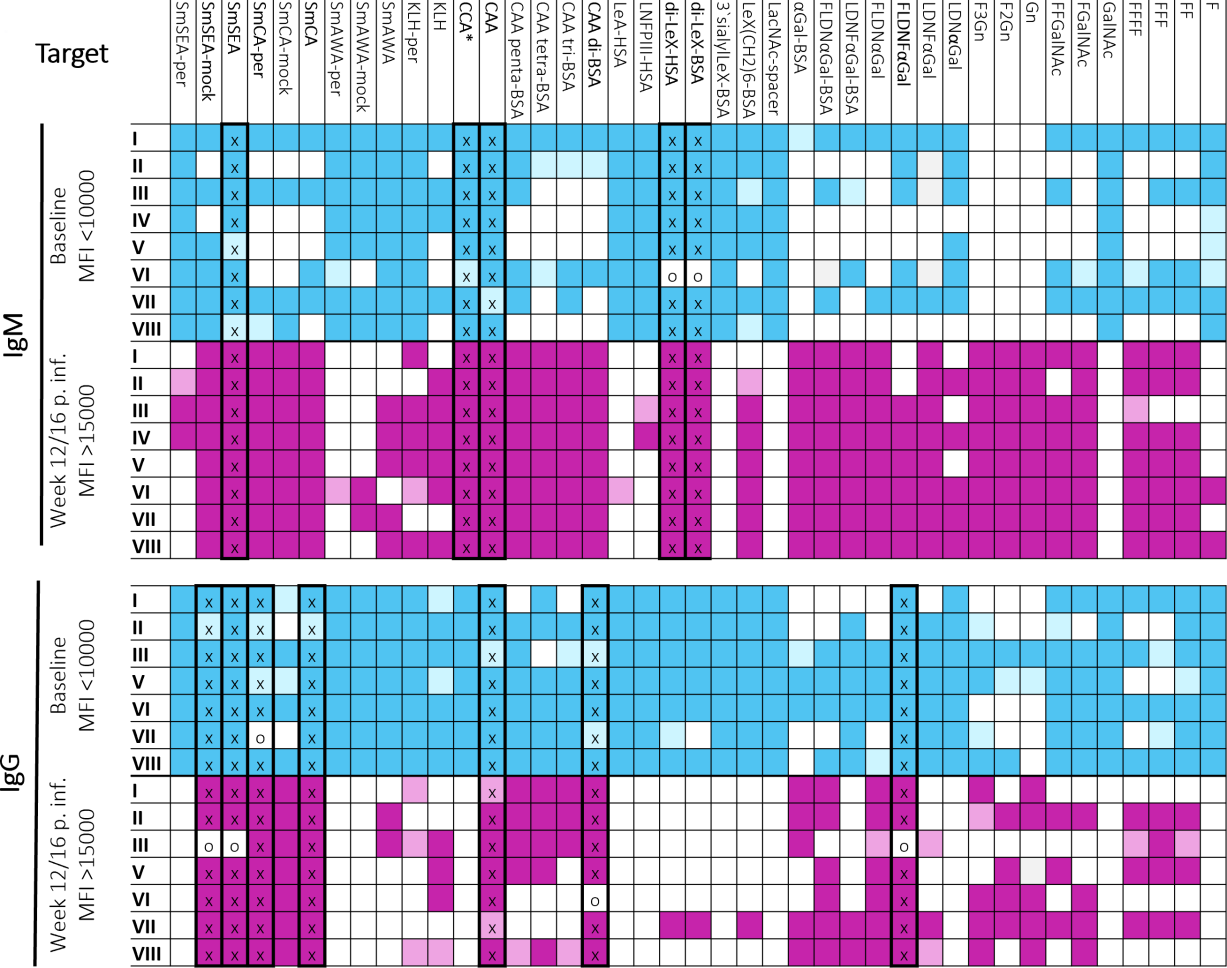
First microarray-based IgM and/or IgG target down-selection by individual level evaluation of antibody responses in controlled human infection samples (I-VIII). Candidate targets were down-selected by a combinatorics approach with requirements for low baseline signal (specificity; MFI<10000) and positive response at week 12 and/or 16 post infection (sensitivity; MFI>15000) measured in CSI1 samples from individuals (I-VIII). Responses between MFI 10000 and 15000 were permitted in both categories for the first down-selection and a single non-complier per category was also allowed, in order to prevent premature exclusion of promising candidates based on a small sample size. Mean fluorescence intensity (MFI) that fulfills criteria: dark blue/purple = yes, no fill = no, light blue/purple = 10000-15000. Thick border around targets fulfilling criteria for all individuals (x = yes, o = one accepted non-responder). p. inf. = post schistosome infection with 20 cercariae. No IgG data for IV available. *All targets were evaluated on the same microarray for all samples except for CCA, which was run on second microarray (Table S1).

Our tailor-made microarrays were combined with well-characterized samples to efficiently assess antibody binding to candidate targets in an iterative manner. The first step in target down-selection from the array panel with relevant crude, purified, and synthetic antigens was done based on a subset of CSI samples. This time-course sample set is ideal for identification of accurate infection-driven antibody responses that are low at baseline (selecting for high specificity) and robustly mounted by all individuals after infection (selecting for high sensitivity). Particularly, CSI samples are suitable to determine specificity cut-offs that are challenging to obtain from cross-sectional or cohort studies in endemic areas due to persistence of antibodies from prior infections. IgM and IgG binding to targets printed on microarrays were evaluated in CSI samples at baseline and 12/16 weeks post-infection (infection dose = 20 cercariae, *n* = 8 [Fig. 1, individuals shown as I–VIII]). Criteria for selecting candidates of interest were low baseline signal (MFI <10,000) and positive response at week 12 and/or 16 post-infection (MFI >15,000). Responses between MFIs of 10,000 and 15,000 were permitted in both categories for the first down-selection, and a single non-complier per category was also allowed. This fluidity in cut-off was considered for first evaluation to avoid prematurely excluding targets of interest based on a small sample size. Targets were considered relevant only if they adhered to both specificity and sensitivity cut-off for IgM and/or IgG. Candidates fulfilling criteria for IgM and/or IgG are shown in bold and with thick border (Fig. 1), and target details can be found in Table S1. For IgM, four targets fulfilled the criteria in the first evaluation: *S. mansoni* soluble egg antigen (SmSEA) and CAA performed with 100% specificity and sensitivity, while di-LeX-HSA and di-Lex-BSA showed 87.5% specificity and 100% sensitivity. For IgG, six targets fulfilled the criteria: SmSEA-mock, SmSEA, di-CAA-BSA, and FLDNFαgal with specificity of 100% and sensitivity of 85.7%, *S. mansoni* cercarial antigen (SmCA)-per with specificity of 85·7% and sensitivity of 100%, while native purified CAA performed the best with 100% for both measures. A second evaluation was done on smaller arrays to reproduce and confirm observations in extended time-course sample series and increased sample size (CSI, *n* = 17). In addition to the synthetic di-LeX element, native CCA was included as this structure is known to consist of repetitive Lewis-X (LeX) motifs (29). The di-CAA element was not included in the second arrays as it is part of the repetitive structure of native purified CAA antigen (30). FLDNFαgal, an abundant motif in the cercarial glycocalyx, which met the performance criteria in the first down-selection, failed with respect to specificity upon assessing more CSI samples (IgM 70.6% [12 of 17] and IgG 58.8% [10 of 17]; see Fig. S1) and is therefore not discussed further.

### Antibodies recognizing crude SmSEA and SmCA show poor specificity on microarray

**Fig 2:**
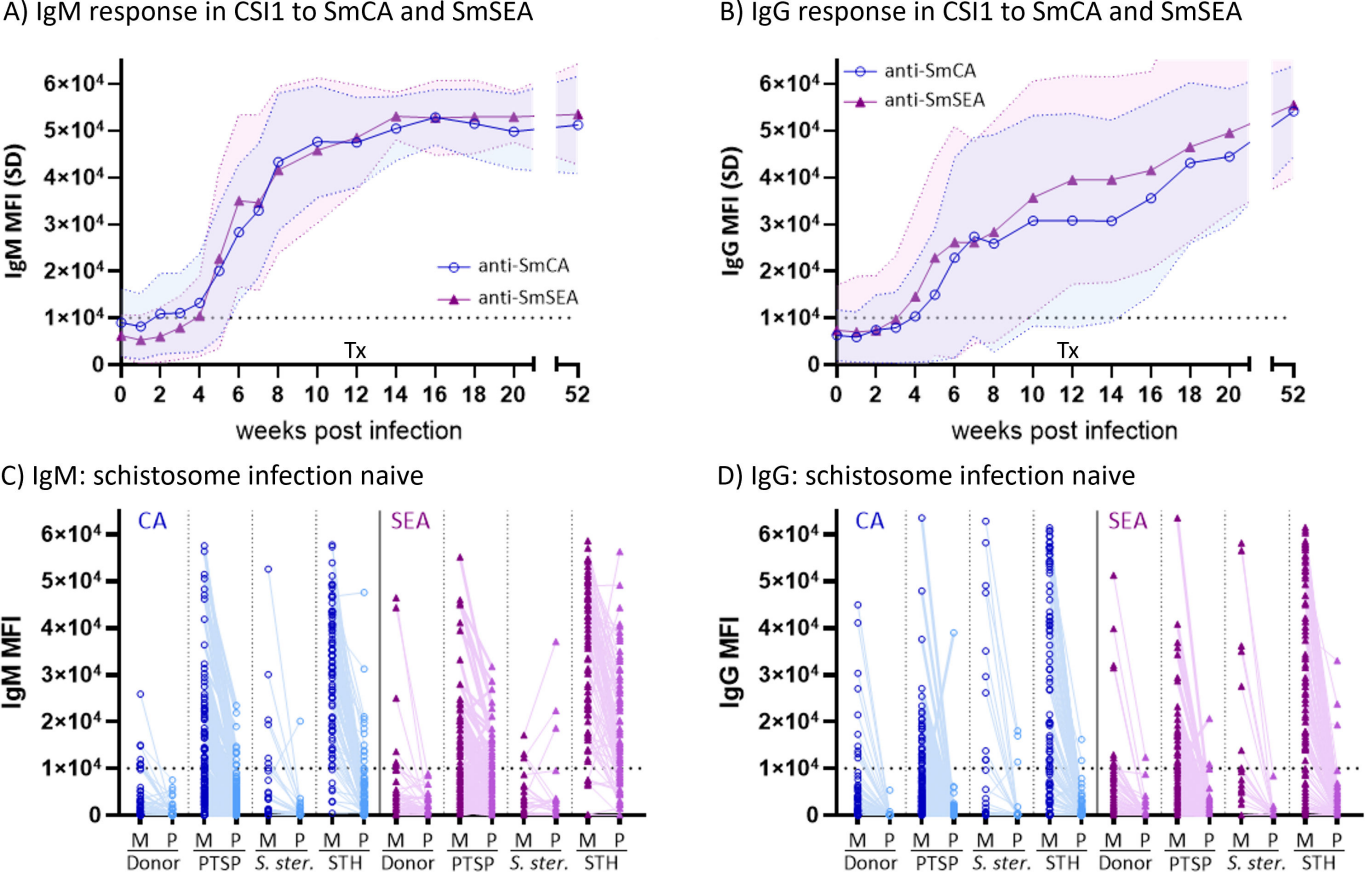
IgM and IgG from individuals with and without schistosome infection recognize glycan epitopes present in *S. mansoni* soluble cercarial (SmCA) and egg antigens (SmSEA). Average SmCA-specific (circles) and SmSEA-specific (triangles) (A) IgM and (B) IgG antibody levels elicited in response to controlled human schistosome infection with male-only cercariae (CSI1: dose 10/20/30 cercariae, *n* = 17 individuals) over time. No parasite eggs were produced in this single-sex CSI model, and hence, all antibodies recognizing SmSEA are binding to shared, predominantly glycan, epitopes present in both SmCA and SmSEA. Tx indicates treatment with 40 mg/kg praziquantel at 12 weeks post infection. (C) IgM and (D) IgG antibodies recognizing SmCA (blue circles) and SmSEA (purple triangles) are present in samples from Dutch blood donors (donor, *n* = 56), from Dutch pre-travel samples (PTSP, *n* = 131), from LUMC biobank samples with *S. stercoralis* infection (*S. ster*., *n* = 25), and from samples with *T. trichiura*, *A. lumbricoides*, and/or hookworm infections from Flores Island, Indonesia, which is also non-endemic for schistosomes (STH IgM, *n* = 87; IgG, *n* = 97). For each individual in each sample set, paired data for mock treated (M, left) and periodate treated (P, right) soluble antigen is shown. A decrease in MFI for the vast majority of antibody binding in periodate compared to mock treated antigen illustrates that many antibodies measured are recongizing glycan elements present within the crude antigen mixes. Such shared glycan epitopes pose a challenge for specificity and hence accuracy in diagnostic immunoassays. See Table S2 for specificity calculations based on the arbitrary cut-off (MFI = 1 × 10^4^) shown as a dotted line on panels C and D. All data shown as mean fluorescence intensity (MFI) were measured on microarray. SD, standard deviation.

Time-course IgM and IgG responses to crude SmCA and SmSEA mixes from baseline to 1 year post-infection in CSI participants present clear infection-related antibody dynamics (Fig. 2A and B). Albeit, variable levels of background reactivity were observed at baseline, which points to a lack of specificity. IgM responses were initiated uniformly upon infection for SmCA and SmSEA, with average seroconversion at week 3 to 4 post-infection (arbitrary cut-off MFI = 10,000). Larger variation for the averaged IgG response curve is explained by more variation in onset of isotype switch from IgM to IgG on an individual level (Fig. 2B). Based on the CSI response, SmCA and SmSEA appear to be good targets for an antibody-detection assay, as they follow a clear dose-response dynamic. However, when measuring anti-SmCA and anti-SmSEA antibodies in samples from schistosome infection-naïve individuals, we found the specificity of these crude antigens to be as low as 8% (IgM) and 31%–36% (IgG) in individuals infected with other helminths (Fig. 2C and D; Table S2). Therefore, these crude antigen preparations are not ideal antibody targets. Much of the reactivity to crude schistosome antigens in schistosome infection-negative individuals can be explained by antibodies recognizing glycan epitopes present in both schistosomes (SmCA and SmSEA) and other organisms. Indeed, antibody binding to these antigens consistently decreased after periodate treatment (Fig. 1, columns 1 and 2; Fig. 2C and D).

### Anti-CAA antibodies show infection dose-dependent isotype switch in primary infection

**Fig 3:**
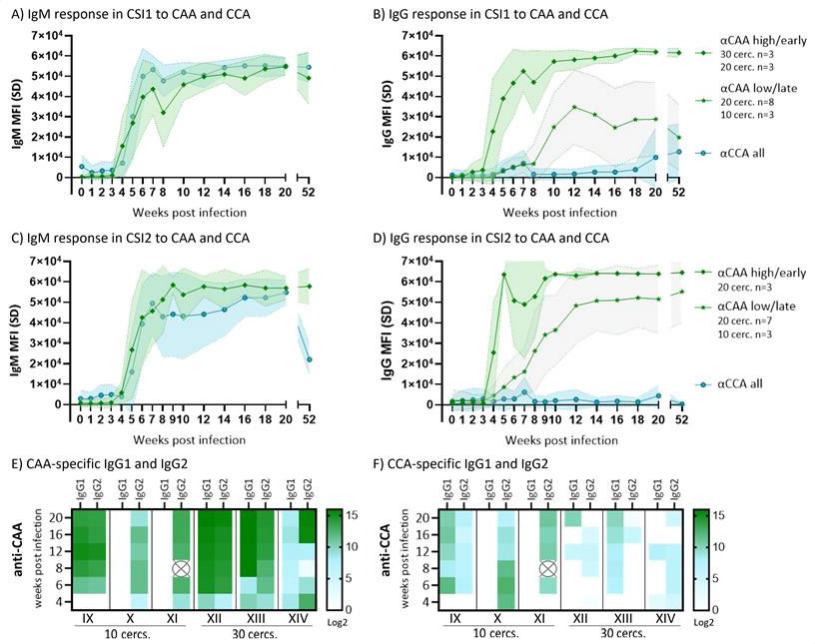
Schistosome circulating anodic and cathodic antigens are immunogenic with IgG response to CAA showing infection dose dependency. Average anti-CAA-specific (rhombes) and anti-CCA-specific (circles) (A) IgM and (B) IgG antibody levels induced in CSI1 with male-only cercariae (dose 10/20/30 cercariae, *n* = 17 individuals) over time at baseline (0) and at 1–8, 10, 12, 14, 16, 18, 20, and 52 weeks post-infection. For panel B, anti-CAA IgG responses are split into two groups depicting individuals responding early (≤week 4, rhombes) and late (≥week 7, stars). The early responder group consisted of 30 (*n* = 3) and 20 (*n* = 3) exposure group individuals, whereas the late responder group consisted of 20 (*n* = 8) and 10 (*n* = 3) exposure group individuals. Area under the curve (AUC × 10^4^) for the early responders is 287.1 (95% CI: 276.1–298.1), and that for the later responders is 111.8 (95% CI: 35.2–188.3). Wilcoxon paired analysis showed that means between the groups over time were significantly different (*P* < 0.0001). (**C**) IgM and (D) IgG responses to CAA and CCA in individuals participating in the CSI2 trial, where they were infected with 10 (*n* = 3) or 20 (*n* = 10) female-only cercariae. Data for baseline (0) and at 1–8, 10, 12, 14, 16, 18, 20, and 52 weeks post-infection are shown. For panel D, IgG responses are split into an early responder group (*n* = 3, 20 cercariae) and a later responder group (*n* = 3, 10 cercariae; *n* = 7, 20 cercariae). AUC × 10^4^ for the early responders is 302.9 (95% CI: 296.2–309.7), and that for the later responders is 232.7 (95% CI: 161.4–303.9). Wilcoxon paired analysis showed significantly different responses between the group means (*p* = 0.0007). The large variation from weeks 5 to 8 in the early responder group in panel D is likely driven by an individual receiving anti-inflammatory treatment (Koopman et al*.* [20]) and the small sample size (*n* = 3). This is less pronounced in panel B due to the larger sample size (Langerberg et al*.* [19]). (A–D) Data shown as MFI with SDs measured on microarray. Note that the signal above 6 × 10^4^ is saturated, and antibody levels may be underestimated when the FI signal is saturated. Heatmap showing (**E**) anti-CAA and (F) anti-CCA IgG1 and IgG2 levels (log2 MFI + 1) for the same six individuals also included in panel B (10 cercs: IX, X, and XI; 30 cercs: XII, XIII, and XIV) at an early phase (weeks 4, 6, and 8) and later phase (weeks 12, 16, and 20) post-infection. No anti-CAA/CCA IgG3 or IgG4 was detectable for IX, X, and XI; IgG4 was not measured for XII, XIII, and XIV; and IgG3 is available in Fig. S2C, where subclass data for anti-SmCA and -SmSEA also can be found (Fig. S2A and B). Any binding below log2 (MFI + 1) = 8 is negligible. ⦻ in heatmap indicates no data available.

Of the defined glycan targets, only the circulating schistosome antigens CAA and CCA fulfilled the first down-selection criteria and upheld performance in the second evaluation of the extended CSI time-course sample set (*n* = 17) (Fig. 1 and 3A and B). CAA is a glycoconjugate containing an antigenic O-linked carbohydrate structure consisting of N-acetyl galactosamine and glucuronic acid (GlcA) repeats [−6(GlcAβ1–3)GalNAcβ1-]n, whereas the CCA structure consists of repeating Lewis-X (LeX) motifs [−3Galβ1–4(Fucα1–3)GlcNAcβ1-]n (29, 30). The assessment of the time-course samples showed that CAA- and CCA-specific IgM was detectable already from 3 to 4 weeks post-infection with comparable dynamics (Fig. 3A). These observations were confirmed by incubation of microarrays with sera from the CSI2 trial, where volunteers were infected with female-only cercariae (Fig. 3C). To CCA, very low IgG titers are elicited in response to infection (Fig. 3B). This low antigenicity is possibly due to epitope similarity between the repetitive LeX motifs of CCA and the LeX antigens present on a subset of human cells and serum glycoproteins (31). This is also consistent with published cross-sectional human data on anti-LeX antibody responses (15). In contrast, anti-CAA IgG responses are much stronger, and isotype switch manifests with an infection dose-dependent timing (Fig. 2B). Individuals exposed to 30 cercariae elicit anti-CAA IgG responses earlier and with higher titers compared to those exposed to only 10 parasites. CSI participants exposed to 20 cercariae can be divided into two groups that follow the anti-CAA IgG response patterns of either the low-dose (10 cercariae) or high-dose (30 cercariae) groups. This corresponds to an early seroconversion of 3 to 4 weeks post-infection in the high dose compared to ≥7 weeks post-infection for the low dose. This dose-dependent delay in isotype switch was confirmed by incubation of sera from the female-only *S. mansoni* infection model (Fig. 3D). Here, three individuals exposed to 20 cercariae elicited early isotype switch, whereas all individuals exposed to the low dose (10 cercariae) and the remainder of those exposed to 20 cercariae isotype switched later albeit marginally earlier than observed in the male-only model. For the current CSI1 and CSI2 participants, the threshold CAA antigen quantity necessary to mount an early response hence falls between infection with 10 and 20 cercariae. The initial IgG subclass repertoire elicited neither depends on antigen dose nor shows change over time (Fig. 3E and F; Fig. S2). However, variation is seen on an inter-individual level in terms of which subclasses are predominantly induced (IgG1, IgG2, or IgG1 + IgG2). Baseline background for both anti-CAA (and CCA) IgM and IgG is markedly lower than that for anti-SmCA and anti-SmSEA, underpinning the high specificity of these antigens Fig. 2A and B, 3A through D.

### Host immune system is exposed to schistosome circulating antigens (CAA and CCA) early after infection

**Fig 4:**
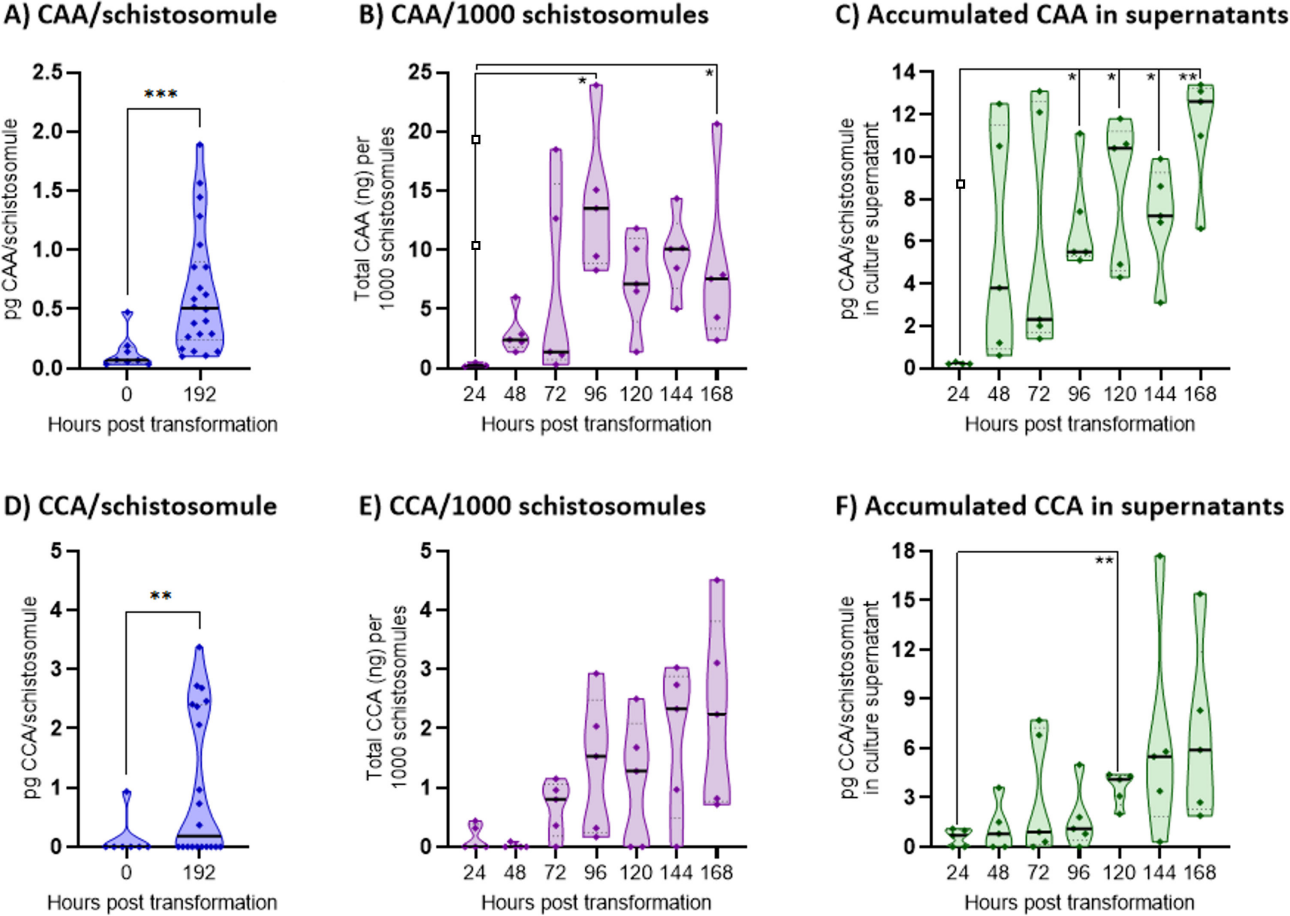
CAA and CCA amounts in schistosomula extracts and excretions. (**A–C**) CAA amount measured by UCP-LF CAA assay and (**D and F**) CCA amount measured by UCP-LF CCA assay shown as truncated violin plots with medians indicated by horizontal full line bars. (**A**) CAA (pg) and (**D**) CCA (pg) measured in schistosomulae individually collected just after transformation from cercariae and 8 days of culturing. Individual pentaplicate wells were harvested from a 48-well culture plate on days 1–7 and 10% vol with parasites counted thrice (parasites/well: range = 761–1,294; mean = 968, median = 977). Transformation efficacy was ≥98%. (**B**) CAA (ng) and (E) CCA (ng) contained in the parasites and adjusted to amount/1,000 schistosomulae at 24-h intervals up to day 7 (168 h). (**C**) Accumulated CAA (pg)/schistosomula and (F) CCA (pg)/schistosomulae excreted into culture supernatants adjusted to parasite count/well. Media controls were included in assays for all time points, and for panels B and C, three datapoints marked with open squares at 24 h are assay-derived technical outliers. The variation in excreted CAA/CCA levels between the five mini-cultures harvested at the same time point is likely due to differences in viability and/or metabolic activity of the schistosomulae despite originating from the same pooled and transformed batch of cercariae. After day 4, it was apparent that some parasites thrived and developed, and others started dying in the culture wells. Hence, the measured CAA/CCA amounts per organism from >96 h is underestimated (**B, C, E, and F**). Note that the technical sensitivity of the UCP-LF CCA assay (buffer format) is lower with a higher cut-off than the CAA assay. Therefore, the low-range values cannot be directly compared between assays and hence the antigens. Welch’s unequal variance *t*-test was used to compare mean CAA/CCA contents in individually collected schistosomulae (**A and D**). Comparison of CAA/CCA content (**B and E**) and excretion (**C and F**) in the multi-well culture experiment was done using Brown-Forsythe and Welch’s ANOVA with Dunnett’s T3 multiple comparisons test.

As specific antibody responses to CAA and CCA were measurable already within 4 weeks post-infection, parasite culture experiments were designed to confirm the presence of these antigens in early life stages. CAA and CCA are gut-associated antigens and likely present in low amounts in the non-functional cercarial gut primordium. Increased expression of these antigens is expected when gut development progresses and the parasites start blood feeding, which also results in antigen regurgitation. To confirm the CAA/CCA presence and increased expression during the course of schistosomulae development, transformed cercariae were cultured and antigen content was measured by UCP-LF CAA and CCA assays. CAA and CCA content was determined in individually collected schistosomulae at days 0 and 8 (Fig. 4A and D). For CAA, we observed a 4.8-fold increase in average CAA/parasite and equivalently a 6.0-fold increase for average CCA/parasite over the eight days. The increase in CAA and CCA produced by schistosomulae was confirmed by measurements done on total schistosomulae harvested from a multi-well experiment on days 1 to 7 as well as the corresponding culture supernatants (Fig. 4B, C, E and F). The antigens are measurable already 24–48 h after culture set-up and reflect a source of antigenic stimuli released into the host circulatory system for uptake and processing.

### Evaluating specificity and sensitivity of anti-CAA and anti-CCA antibodies

**Fig 5:**
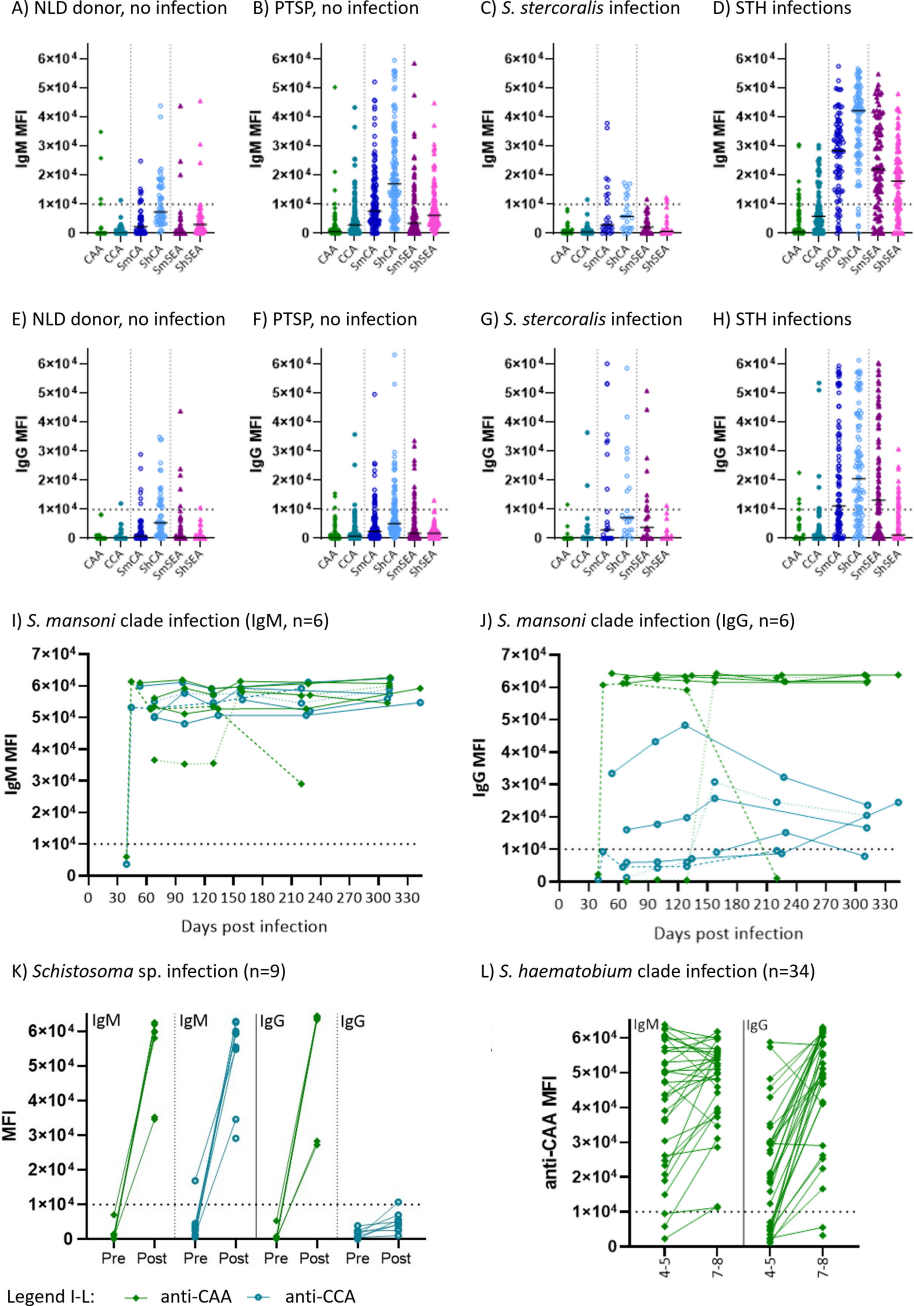
IgM and IgG antibodies recognizing CAA are highly specific and sensitive. Samples from schistosomiasis non-endemic areas were used to assess specificity of antibodies binding to circulating schistosome antigens compared to crude antigen preparations (**A–H**). IgM (**A–D**) and IgG (**E–H**) responses to CAA, CCA, *S. mansoni* CA (SmCA), *S. haematobium* CA (ShCA), *S. mansoni* SEA (SmSEA), and *S. haematobium* SEA (ShSEA) for schistosomiasis infection-naïve individuals are shown as dotblots for Dutch blood donors (A and E, *n* = 56 NLD donor), Dutch pre-travel samples (B and F, *n* = IgM 130/IgG 131 PTSP), *S. stercoralis*-infected LUMC biobank samples (C and H, *n* = 25), and soil-transmitted helminth (*T. trichiura*, *A. lumbricoides*, and/or hookworm)-infected individuals from Pulau Flores, Indonesia (D and H, *n* = IgM 93/IgG 97 STH) (27). See Table 1 for specificity calculations based on the arbitrary cut-off (MFI = 10,000) shown as dotted lines on the graphs (**A–H**). Primary schistosome infection traveler samples were used to confirm sensitivity (**I–L**) of anti-CAA and anti-CCA antibodies. IgM (**I**) and IgG (**J**) responses to CAA and CCA in six young male Spanish travelers exposed to *S. mansoni* clade parasite in Chicamba Lake, Mozambique, in 2019 through a single swimming activity (24). Individuals shown with dashed lines are paired for panels I and J to highlight time dynamics for individuals without consistently high antibody titer. These two individuals show differences in timing of isotype switch. One individual was the first to present with symptoms at the travel clinic at day 39 after infection (low antibodies) and with high IgM and IgG at 44 days post-infection and declining antibody levels from ~20 weeks post-infection. The second has lower IgM from first measurement at ~7 up to ~20 weeks post-exposure, where isotype switch occurs and CAA-specific IgG is induced. (**K**) Anti-CAA and anti-CCA for paired pre- and post-travel samples from nine adult Dutch individuals all identified as schistosomiasis IgM positive but only three were IgG positive in the original study (21). These nine individuals were exposed to *Schistosoma* sp. through swimming in Lake Malawi and/or Lake Victoria at separate events during travel of varying lengths and measured within 3 months after returning to the Netherlands. All post-travel samples from individuals with no schistosome exposure were confirmed to still be negative for anti-CAA and anti-CCA (PTSP pre-travel shown in panels B and F). (**L**) Anti-CAA responses for 34 Belgian individuals traveling as part of two separate groups of families but exposed at the same location through swimming and rafting in uMhkunyane river, Northern Kwa-Zulu Natal, South Africa, in 2016/2017 to *S. haematobium* clade parasites (*S. haematobium* × *S. mattheei* hybrid and/or co-infection(s)) within a 2-week interval (25, 26). No data for anti-CCA antibodies were available for this sample set due to limited sample volume. All data shown as MFI measured on microarray with medians indicated as horizontal bars.

To further evaluate specificity of CAA and CCA as antibody targets compared to crude schistosome antigen preparations, more samples from schistosomiasis-naïve individuals were assessed: firstly, we analyzed samples from individuals unlikely to have been exposed to or infected with helminths (Dutch blood donors and pre-travel study samples). Secondly, we analyzed samples from individuals with soil-transmitted helminth infections. Schistosomiasis false positives for both IgM and IgG are much less frequent for CAA and CCA compared to crude antigen from both *S. mansoni* and *S. haematobium* (Fig. 5A through H, same arbitrary cut-off as Fig. 2). The superior specificity of CAA and CCA compared to crude SmCA and SmSEA is outlined in Table 1. IgG performs with ≥96% specificity for anti-CAA and ≥92% for anti-CCA across all sample sets, whereas the specificity falls as low as 31% for anti-ShCA and 40% for anti-SmSEA in the STH-infected individuals. Specificity for IgM is slightly lower across sample sets with the lowest anti-CAA performance for STH samples at 87% and anti-CCA at only 73%. However, for crude antigens, specificity in STH-infected individuals is between 6% and 30%, highlighting the gross cross-reactivity mediated by shared epitopes. This low specificity renders the crude antigen mixes useless in diagnostic context when measured on microarray. Three sample sets from Europeans who were infected during travel in schistosomiasis endemic areas were used to further investigate sensitivity of anti-CAA and anti-CCA antibodies. High IgM and IgG antibody levels in six young Spanish travelers were seen at ≥44 days post-infection (Fig. 5J). All six individuals have high anti-CAA and anti-CCA IgM levels, whereas IgG is high for anti-CAA and less pronounced for anti-CCA, consistent with CSI observations (Fig. 3B). A similar pattern was observed in samples from a historical pre- and post-travel study that included nine schistosomiasis cases (Fig. 5K). Importantly, anti-CAA IgG performed better than the SEA-ELISA and/or IFA (adult worm sections) used in the original study, which consisted of data from travel clinics (nine of nine compared to three of nine). A third primary infection sample set consisted of travelers exposed to *S. haematobium* clade parasites. Anti-CAA antibodies were measurable in 34 of 34 for IgM and 32 of 34 for IgG at 7–8 weeks post-infection (Fig. 5L). This finding is consistent with the cumulative positive pre-treatment serum CAA findings (33 of 34 positive) previously published (26). Merging the CSI and primary infection traveler sample sets gives an overall sensitivity for anti-CCA IgM of 100% (IgG not usable) and for anti-CAA IgM of 100%; for IgG of 97% (*n* = 66, arbitrary cut-off MFI = 10,000, timing variable). These three primary infection traveler sample sets confirm the high sensitivity of anti-CAA and -CCA antibodies observed in CSI (Fig. 3A and B).

**TABLE 1:** Specificity of CAA, CCA, and crude *S. mansoni* and *S. haematobium* antigens on microarray^*a*^^*,*^^*b*^

Specificity %^*c*^ (*n* true negatives/*n*)	CAA	CCA	SmCA	ShCA	SmSEA	ShSEA
IgM (all samples)	**93.4** (**284/304**)	84.9 (258/304)	50.3 (153/304)	30.7 (89/303)	66.8 (203/304)	64.1 (195/304)
Donor (no. inf)	92.9 (52/56)	98.1 (55/56)	83.9 (47/56)	58.9 (33/56)	94.6 (53/56)	94.6 (53/56)
PTSP (no. inf)	96.9 (126/130)	85.4 (111/130)	58.5 (76/130)	27.7 (36/130)	80 (104/130)	70.8 (92/130)
*S. ster*.	100 (25/25)	96 (24/25)	64 (16/25)	72 (18/25)	96 (24/25)	88 (22/25)
STH	87.1 (81/93)	73.1 (68/93)	15.1 (14/93)	6.5 (6/92)	23.7 (22/93)	30.1 (28/93)
IgG (all samples)	**97.4** (**301/309**)	**95.8** (**296/309**)	73.1 (226/309)	57.9 (179/309)	71.8 (222/309)	**92.9** (**287/309**)
Donor (no. inf)	100 (56/56)	98.1 (55/56)	89.3 (50/56)	64.3 (36/56)	91.1 (51/56)	98.1 (55/56)
PTSP (no. inf)	97.7 (128/131)	97.7 (128/131)	87.0 (114/131)	73.3 (96/131)	87.8 (115/131)	99.2 (130/131)
*S. ster*.	96 (24/25)	92.0 (23/25)	68 (17/25)	68 (17/25)	68 (17/25)	96 (24/25)
STH	95.9 (93/97)	92.8 (90/97)	46.4 (45/97)	30.9 (30/97)	40.2 (39/97)	80.4 (78/97)

^*a*^ Sample sets: donor (no. inf), no infection (G); PTSP, post-travel screening of parasites (B); *S. ster.*, *Strongyloides stercoralis* (F); STH, soil-transmitted helminth (E).

^*b*^ Specificities above 90% are indicated with gray shading (sample sets) or bold font (total).

^*c*^ Based on arbitrary cut-off at MFI = 10,000.

## DISCUSSION

Through the microarray-based evaluation of a panel of representative schistosome antigens, CAA emerged as the best-performing target with overall high sensitivity (IgM ≥100% and IgG ≥97%) and specificity (IgM ≥93% and IgG ≥97%) for serological detection of primary schistosomiasis infection. The high specificity of the existing CAA antigen detection assay supports the observation of superior specificity of anti-CAA antibodies as primary infection marker (19, 32). Data on characteristics of antibody responses to schistosome infection such as isotype, IgG subclass, induction timing, and persistence inform how and when serological tests can be used optimally within diagnostic algorithms for different epidemiological settings. We envision use cases for anti-CAA antibody detection in non-endemic area travel medicine (IgG and/or IgM) and as a surveillance tool (IgG). For example, detection of highly specific antibodies in people exposed to fresh water bodies in areas with risk of transmission (re-)emergence such as the Mediterranean and/or climate change-mediated risk zones can be a cost-effective first-line surveillance tool (33). Anti-CAA antibody detection is expected to be pan-schistosome species specific, as the antigen has been shown to be excreted by several schistosome species (34–37). Evaluation with well-characterized samples from people infected with other schistosome species such as *S. japonicum*, *S. haematobium*, *S. mekongi*, *S*. *intercalatum*, and hybrid species/co-infections can increase the applicability of future antibody-detection tests even further. Our starting CSI sample set for target down-selection, favors immunogenic antigens expressed by cercariae, juvenile, and adult schistosomes. Therefore, it cannot be excluded that antigens expressed by eggs also could perform well. However, our candidate selection process and antigen portfolio are excellent for our envisioned use cases targeting primary and relatively recent primary infection in line with the WHO diagnostic target product profiles (TPPs) (3).

Interestingly, we observed an infection dose-dependent isotype switch in CSI time-course samples. IgM responses to CAA were on average detectable already at 3 to 4 weeks post-infection irrespective of infection dose. However, isotype switch to IgG varied in an infection dose-dependent manner with a 3- to 4-week delay for individuals infected with a low dose (10 cercariae) compared to those with a higher dose (30 cercariae). Measuring total antigen-specific IgG is advantageous compared to detection of a single subclass, as not all individuals elicit the same subclass to CAA as observed in the CSI samples (IgG1 or IgG2 or IgG1/IgG2). In this study, no IgG3 and IgG4 recognizing CAA or glycan elements contained in crude SmCA or SmSEA were detectable in CSI samples. However, both IgG3 and IgG4 specific for epitopes such as core-xylose elements present in crude antigen have been demonstrated in chronic infection samples (38, 39). Limited data from older studies support the antigenicity of the unique polysaccharides, CCA and CAA, expressed in the schistosome gut. In concordance with our anti-CAA and anti-CCA observations, Nash et al. (22) showed by IFA that individuals with acute infection presented with high antibody levels to gut-associated polysaccharides, now known to include CCA and CAA (22). In contrast, chronic infection antibody levels were lower and disconnected from egg burden with increasing age (22, 40). It is still unknown under which infection pressures down-modulation and/or immune-complex formation may result in lower CAA-specific IgM and IgG measurements (41). Nevertheless, anti-CAA antibody detection can be a powerful surveillance tool in near- and post-elimination settings if applied in defined target groups such as occupational risk groups or children born after reaching intervention goals and hence essentially targeting recent primary infection cases.

The current WHO roadmap and importantly the TPP (2, 3) highlight the need for accurate diagnostic tools and mapping to define intervention implementation units and target limited resources, where they have the most impact. As outlined in the TPP, antibody-detection tools are useful for surveillance in very low-prevalence settings where there are less people with prior infection, particularly in the younger age groups. Furthermore, such tools are useful as indicators for when to stop mass drug administration and apply test-then-treat approaches. In this scenario an antibody-detection test could function as the first-line tool followed by a molecular or antigen detection tool as second-line confirmation of current active infection. Specifically, antibody-detection tools fit into the TPP to determine when transmission has been interrupted and when to conduct post-mass drug administration (MDA) surveillance (3). Ideally, a rapid-diagnostic finger-prick anti-CAA test format is developed based on detection of IgG as this shows the best specificity. However, depending on screening needs, an IgM or total immunoglobulin (including IgA and IgE, if present) test could be considered, especially in settings where a second-line current infection tool is applied. Using a highly specific defined antigen target such as CAA also confers the advantage of potential incorporation into multiplexed multi-disease (bead-based) diagnostic assays, which can be cost-effective for laboratory-based sentinel surveillance.

For upscaled and quality-controlled production of a serological test, whether in finger-prick rapid-diagnostic lateral flow test, ELISAs (direct/sandwich and or competition), immunoblot, or bead/nanoparticle-based (multiplex) platform formats, the target must be produced in validated and feasible manners. This is achievable for relatively low throughput in house assays based on immunopurified native CAA such as formats used for non-endemic area travel medicine and research. Development of tests aimed at upscaled serosurveillance or commercial formats will require establishment of chemical, enzyme-assisted, or biotechnological synthesis of the catching antigen, CAA. While exogenous carbohydrate antigens are challenging to express in well-established systems, whether bacterial, yeast, baculoviral, or mammalian cell line based, promising alternative platforms engineered for glycan antigen production are emerging. Successful glycoengineering of the N-glycosylation machinery in *Nicotiana benthamiana* plants and production of glycoproteins with defined helminth-associated elements show that it is possible to recapitulate and produce complex glycan structures from schistosomes (42) when the corresponding components of biosynthetic machinery have been identified. In the case of CAA, which is a carbohydrate structure consisting of N-acetylgalactosamine (GalNAc) and glucoronic acid (GlcA) repeats (30), that would be the specific β6GalNAc-transferase and β3GlcA-transferase. Basing a test on carbohydrate rather than protein catching antigen encompasses the added value of better temperature stability and no vulnerability to protease degradation. The native CAA antigen has been found in more than 3,000-year-old mummies and is known to be highly stable at room temperature, refrigerated, frozen, and in lyophilized form (43, 44). Such properties mean potential for less dependency on cold-chain transport and storage, which are factors that can have significant impact on cost and feasible real-life use in resource-poor areas. In conclusion, this study has identified CAA as an antibody target with promising accuracy for (recent) primary schistosomiasis infection. Development of a highly sensitive and specific antibody-detection assay will be a beneficial addition to the existing diagnostic tool repertoire for schistosomiasis. An anti-CAA antibody-detection tool would have a particular impact and use in traveler diagnostics and for surveillance in near- and post-elimination, eradication, and emerging transmission zone settings.

## Data Availability

Microarray data are available in the Mendeley repository under the following DOI: 10.17632/r2bj3nm82f.1.

## SUPPLEMENTAL MATERIAL

Supplemental material jcm.01008-24-s0001.pdf Supplemental methods, Tables S1 and S2, and Fig. S1 and S2.

ASM does not own the copyrights to Supplemental Material that may be linked to, or accessed through, an article. The authors have granted ASM a non-exclusive, world-wide license to publish the Supplemental Material files. Please contact the corresponding author directly for reuse.
