# Supplementary material for: Identification of a circulating carbohydrate antigen as a highly specific and sensitive target for schistosomiasis serology
Source: J Clin Microbiol. 2025 Jan 13;63(2):e01008-24. doi: 10.1128/jcm.01008-24 (PMC11837524; doi:10.1128/jcm.01008-24)
Supplement: Supplemental material — Supplemental methods, Tables S1 and S2, and Fig. S1 and S2. [file jcm.01008-24-s0001.pdf]

## SUPPLEMENTARY for "Identification of a circulating carbohydrate antigen as a highly specific and sensitive target for schistosomiasis serology, Kildemoes *et al.* 2024"

## SECTION 1: METHODS

***Additonal methodology for microarray construction and incubation with anonymised serum/plasma samples***

**Construction of microarrays**

Crude schistosome soluble cercarial antigen and egg antigen from both *S. mansoni* (SmCA, SmSEA) and *S. haematobium* (ShCA, ShSEA) was produced by mechanical disruption of parasite material in cold PBS followed by sonification (Branson Sonic Power Company, Sonifier B-12) and centrifugation (17000g). The material was split for sodium meta-periodate ($NaIO_4$) and mock treatment for glycan epitope disruption. Mock treatment and $NaIO_4$ treatment were done in pre-cooled 0.4 M acetate buffer (pH 4.5, NaAc3H2O, JTBaker, 0256) with and without 40 mM of $NaIO_4$ (Merck, 1.0659) respectively (o/n cold incubation on roller). Quenching was done with one volume of 50 mM sodium borohydride ($NaBO_4$, Fluka, 71321) for 30 minutes on ice. All material was dialysed against 1x PBS o/n (Thermo-Scientific, Slide-a-Lyzer mini dialysis devices 3500MWCO).
Custom arrays were setup in 384 well plates (Greiner bio-one, 784201). These arrays contained synthetic glycan elements, keyhole limpet haemocyanin (Sigma-Aldrich, H7017), native schistosome circulating cathodic and anodic antigens (CCA, CAA), printbuffer (Nexterion Spot 1066029) controls, and crude schistosome antigen preparations with and without $NaIO_4$ treatment. All antigens were prepared in 10% DMSO (Sigma-Aldrich D8418) in printbuffer and printed in triplicates/array onto epoxilane coated glass slides (Nexterion Slide E, Schott 1066643) with a MicroGrid robot (BioRobotics) (1). Full target list and details can be found in Table S1.

**Microarray incubations**

Microarrays with fitted silicone gaskets enabling incubation with 8 or 64 samples were reconstituted in PBS and blocked for a minimum of one hour (PBS, 2% bovine serum albumin (Sigma-Aldrich A3059) with 50mM ethanolamine (>99.5%, Sigma-Aldrich 411000) on shaker. Each array was washed in PBS-Tween0·05%, PBS and 1:100 serum sample in buffer/array added for 1 hour incubation at room temperature while shaking. For 8 sample gaskets a total of 250 µl diluted sample/array was used whereas 30 µl/array was used for 64 sample gaskets. Arrays were then washed (as above) and 1:1000 detection antibodies added (goat anti-human IgG-Cy-3, Sigma-Aldrich C2571; goat anti-human IgM-AF647, Invitrogen, A21249) for 30 min room temperature incubation on shaker. For IgG subclass experiments serum samples were measured in 1:50 dilutions and as detection antibodies the following dilutions and antibodies were used; 1:400 mouse anti-human (Southern Biotech) IgG1 Hinge-AF555 (#9052-32), IgG2 Fc-AF555 (#9070-32), and IgG3 Hinge-AF647 (#9210-31) were used and IgG4 pFc'-AF647 (#9190-31) in 1:250. Finally, slides were washed as above with an additional wash in milli-Q before they were spun dry and scanned (Agilent Scan Control™ 2006). The scanner can accommodate two fluorophores per microarray, so IgG/IgM or pairs of IgG subclasses were measured in the same experiments

**Schistosomula culture and UCP-LF CAA and CCA measurements**

*S. mansoni* cercariae were transformed by heat-shock. Briefly cercariae were shed from *Biomphalaria glabrata* snails in Bar-Le Duc water and then kept on ice for ~two hours. Prewarmed culture media (Hybridoma-SFM (Gibco ref. 12045-076), Penicillin-streptomycin (Gibco ref. 15140122), 200 µM ascorbic acid (J.T. Baker ref. 1018), 1:500 chemically defined lipid concentrate (Gibco ref. 11905031)) was added to sedimented cercariae. Incubation was done in 37℃ waterbath with mechanical manual mixing every five minutes for 20 minutes. Schistosomulae were separated from tails on orbital shaker and transferred for 20 minutes, 37℃ incubation with antibiotics and antimycotic (1:100 ABAM (Sigma-Aldrich ref. A5955) in Dulbecco's PBS (DPBS, Sigma-Aldrich D8662). Schistosomulae were sedimented and media changed to 10 ml media with 0 · 02% red blood cells (Sanquin, washed in RPMI1640 HEPES, Gibco ref. 13018015) added for plating into 48 well plate (200 µl parasites plus 800 µl media/well). Single schistosomulae baseline and day 8 samples were collected. For day 1-7 five full well replicates and a control were collected. Parasites were counted in 100 µl per well at 2x magnification. The remaining material was split into supernatant and parasite material by centrifugation at 2000g. Parasite pellets were washed twice in DPBS and subsequently freeze-dried, resuspended in DPBS and sonified. CAA and CCA were measured in supernatant and schistosomulae by UCP-LF BCAAhT17 and UCP-LF BCCAhT17 wet assays, respectively (urine assay protocols (2) with urine to PBS adaptation and 2% final concentration TCA). Cut-offs that apply to these culture media experiments are: UCP-LF BCAAhT17 = 10 pg/ml, UCP-LF BCCAhT17 = 1000 pg/ml (2, 3).

Table S1: Microarray target list for first down-selection arrays (8 samples gasket)

| Abbreviation | Structure/full name | Linker/protein carrier | Comment | Material origin (reference) |
|---|---|---|---|---|
| F | Fucα1- | 6-aminohexan-1-ol linker | synthetic | Harvey *et al.* (4) |
| FF | Fucα1-2Fucα1- | 6-aminohexan-1-ol linker | synthetic | Harvey *et al.* (4) |
| FFF | Fucα1-2Fucα1-2Fucα1- | 6-aminohexan-1-ol linker | synthetic | Harvey *et al.* (4) |
| FFFF | Fucα1-2Fucα1-2Fucα1-2Fucα1- | 6-aminohexan-1-ol linker | synthetic | Harvey *et al.* (4) |
| GalNAc | GalNAcβ1- | 6-aminohexan-1-ol linker | synthetic | Harvey MR (5) |
| FGalNAc | Fucα1-3GalNAcβ1- | 6-aminohexan-1-ol linker | synthetic | Harvey MR (5) |
| FFGalNAc | Fucα1-2Fucα1-3GalNAcβ1- | 6-aminohexan-1-ol linker | synthetic | Harvey MR (5) |
| Gn | GlcNAcβ1- | 6-aminohexan-1-ol linker | synthetic | Van Roon *et al.* (6) |
| F2Gn | Fucα1-2Fucα1-3GlcNAcβ1- | 6-aminohexan-1-ol linker | synthetic | Van Roon *et al.* (6) |
| F3Gn | Fucα1-2Fucα1-2Fucα1-3GlcNAcβ1- | 6-aminohexan-1-ol linker | synthetic | Van Roon *et al.* (6) |
| LDNaGal | GalNAc-β-(1- 4)-GlcNAcβ1-3Galα1- | 5-aminopenta-1-ol linker | synthetic | Ágoston *et al.* (7) |
| LDNFaGal | GalNAcβ1-4(Fucα1-3)GlcNAcβ1-3Galα1- | 5-aminopenta-1-ol linker | synthetic | Ágoston *et al.* (7) |
| FLDNFaGal | Fucα1-3GalNAcβ1-4(Fucα1-3)GlcNAcβ1-3Galα1- | 5-aminopenta-1-ol linker | synthetic | Ágoston *et al.* (7) |
| FLDNaGal | Fucα1-3GalNAcβ1-4GlcNAcβ1-3Galα1- | 5-aminopenta-1-ol linker | synthetic | Ágoston *et al.* (7) |
| LDNFaGal-BSA | GalNAcβ1-4(Fucα1-3)GlcNAcβ1-3Galα1- | bovine serum albumin | synthetic | Ágoston *et al.*,van Remoorte *et al.* (7, 8) |
| FLDNaGal-BSA | Fucα1-3GalNAcβ1-4GlcNAcβ1-3Galα1- | bovine serum albumin | synthetic | Ágoston *et al.*,van Remoorte *et al.* (7, 8) |
| aGal-BSA | Galα1-3Galβ1-4GlcNAcβ1- | bovine serum albumin | Commercial synthetic | Dextra Laboratories Ltd (UK) |
| LacNAc( | Galβ1-4GlcNAcβ1- | bovine serum albumin | synthetic | Van Roon *et al.* (9, 10) |
| LeX-BSA | Galβ1-4(Fucα1-3)GlcNAcβ1 | bovine serum albumin | synthetic | Van Roon *et al.* (9, 10) |
| 3`sialylLeX-HSA | NeuAcα2-3Galβ1-4(Fucα1-3)GlcNAcβ1 | bovine serum albumin | Commercial synthetic | Isosep (Tullinge, Sweden) |
| di-LeX-BSA | Galβ1-4(Fucα1-3)GlcNAcβ1-3Galβ1-4(Fucα1-3)GlcNAcβ1- | bovine serum albumin | synthetic | Van Roon *et al.* (9, 10) |
| di-LeX-HSA | Galβ1-4(Fucα1-3)GlcNAcβ1-3Galβ1-4(Fucα1-3)GlcNAcβ1- | human serum albumin | Commercial synthetic | Isosep (Tullinge, Sweden) |
| LNFPIII-HSA | Galβ1-4(Fucα1-3)GlcNAcβ1-3Galβ1-4Glcβ1- | human serum albumin | Commercial synthetic | Isosep (Tullinge, Sweden) |
| LeA-HSA | Galβ1-3(Fucα1-4)GlcNAcβ1 | human serum albumin | Commercial synthetic | Isosep (Tullinge, Sweden) |
| CAA di-BSA | GlcAβ1-3GalNAcβ1- | bovine serum albumin | synthetic | Halkes *et al.*, Vermeer *et al.* (11, 12) |
| CAA tri-BSA | GalNAcβ1-6(GlcAβ1-3)GalNAcβ1- | bovine serum albumin | synthetic | Halkes *et al.*, Vermeer *et al.* (11, 12) |
| CAA tetra-BSA | GlcAβ1-3GalNAcβ1-6(GlcAβ1-3)GalNAcβ1- | bovine serum albumin | synthetic | Halkes *et al.*, Vermeer *et al.* (11, 12) |
| CAA penta-BSA | GalNAcβ1-6(GlcAβ1-3)GalNAcβ1-6(GlcAβ1-3)GalNAcβ1- | bovine serum albumin | synthetic | Vermeer *et al.* (12) |
| CAA | [-6(GlcAβ1-3)GalNAcβ1-]n | none | *S. mansoni,* Immunopurified, capture antibody 51-4G5-A | van Dam *et al.* (13) |
| KLH | Hemocyanin | none | commercial, from *Megathura crenulata* | Sigma-Aldrich, H7017 |
| KLH-per | Hemocyanin sodium meta-periodate treated | none | commercial, from *Megathura crenulata* | Sigma-Aldrich, H7017 |
| SmAWA | adult worm antigen | none | mix of crude soluble antigens | *S. mansoni* LUMC life cycle* |
| SmAWA-mock | adult worm antigen mock treated | none | mix of crude soluble antigens | *S. mansoni* LUMC life cycle* |
| SmAWA-per | adult worm antigen sodium meta-periodate treated | none | mix of crude soluble antigens | *S. mansoni* LUMC life cycle* |
| SmCA | cercarial antigen | none | mix of crude soluble antigens | *S. mansoni* LUMC life cycle* |
| SmCA-mock | cercarial antigen mock treated | none | mix of crude soluble antigens | *S. mansoni* LUMC life cycle* |
| SmCA-per | cercarial antigen sodium meta-periodate treated | none | mix of crude soluble antigens | *S. mansoni* LUMC life cycle* |
| SmSEA | egg antigen | none | mix of crude soluble antigens | *S. mansoni* LUMC life cycle* |
| SmSEA-mock | egg antigen mock treated | none | mix of crude soluble antigens | *S. mansoni* LUMC life cycle* |
| SmSEA-per | egg antigen sodium meta-periodate treated | none | mix of crude soluble antigens | *S. mansoni* LUMC life cycle* |

| | | | | |
|---|---|---|---|---|
| printbuffer | Proprietary | none | Commercial | Schott, Nexterion Spot 1066029 |
| Second round reproduction and confirmation arrays (64-gasket) included the targets below as well as all targets above with promising performance in first run. | | | | |
| CCA* | [-3Galβ1-4(Fucα1-3)GlcNAcβ1-]n | none | *S. mansoni*, , immunopurified, capture antibody 54-5C10-A | van Dam *et al.* (13) |
| ShCA | cercarial antigen | none | mix of crude soluble antigens | *S. haematobium* LUMC life cycle^ |
| ShSEA | egg antigen | none | mix of crude soluble antigens | *S. haematobium* LUMC life cycle^ |
| HSA | Human serum albumin | none | commercial | Sigma-Aldrich |
| **Schistosoma mansoni* is the Puerto Rican strain and propagated at LUMC since 1955 (14).<br>^*Schistosoma haematobium* is from NAMRU-3, Cairo, Egypt propagated at LUMC since ~1993 | | | | |

## SECTION 2: RESULTS

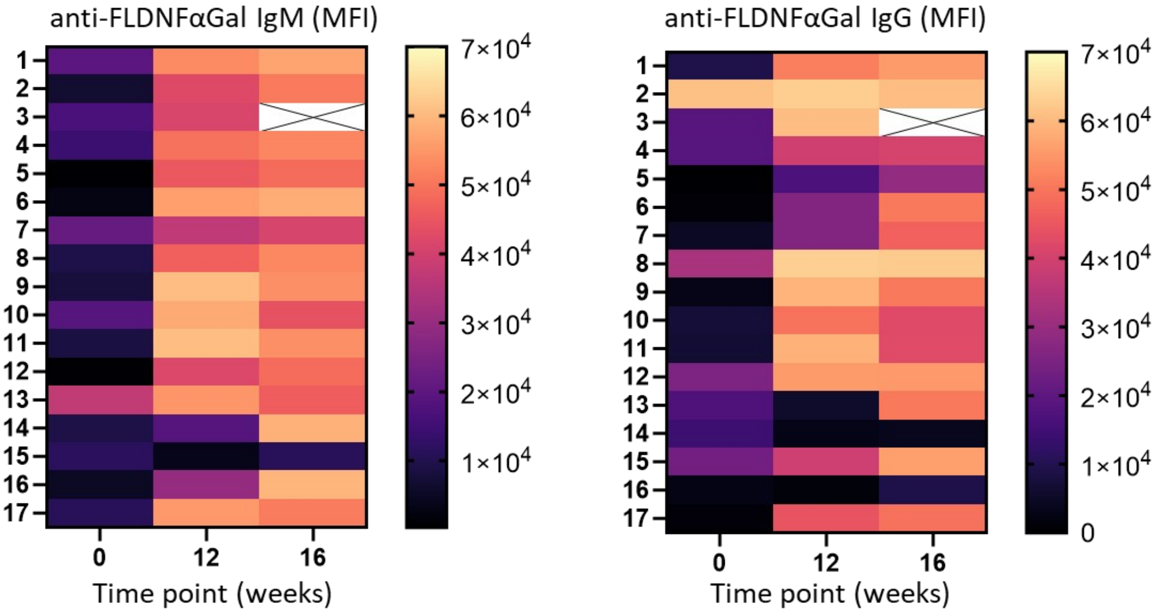


**Fig S1: anti-FLDNFαgal IgM and IgG reproduction and confirmation arrays**

Heatmaps showing anti-FLDNFαgal IgM (left) and IgG (right) responses at baseline (week 0) and week 12/16 post infection with male *S. mansoni* cercariae for CSI participants (n=17). FLDNFαgal met the performance criteria in the first down-selection (n=7 CSI1) but failed with respect to specificity upon assessing more CSI1 samples in this second round of reproduction and n increase to confirm candidate target performances. Specificity with a cut-off at MFI=15000: IgM 70·6% (12/17); IgG 58·8% (10/17). Specificity with a cut-off at MFI=10000: IgM 52·9% (9/17); IgG 52·9% (9/17). Sensitivity (week 12/16) with a cut-off at MFI=15000: IgM 94·1% (16/17); IgG 88·2% (15/17). Sensitivity (week 12/16) with a cut-off at MFI=10000: IgM 100% (17/17); IgG 88·2% (15/17). X in heatmap = no data available.

**Table S2: Shared glycan epitopes present in soluble crude SmCA and SmSEA decrease specificity**

| ***IgM specificity*** | Donor* | PTSP* | *S. ster.** | STH* |
|---|---|---|---|---|
| SmCA-mock | 83·9 (47/56) | 57·7 (75/130) | 72·0 (18/25) | 8·0 (7/87) |
| SmCA-periodate | 100 (56/56) | 90·0 (117/130) | 96·0 (24/25) | 86·2 (75/87) |
| SmSEA-mock | 87·5 (49/56) | 63·8 (83/130) | 84·0 (21/25) | 8·0 (7/87) |
| SmSEA-periodate | 100 (56/56) | 80·0 (104/130) | 88·0 (22/25) | 36·4 (30/87) |
| ***IgG specificity*** | | | | |
| SmCA-mock | 82·1 (46/56) | 74·8 (98/131) | 60·0 (15/25) | 36·1 (35/97) |
| SmCA-periodate | 100 (56/56) | 99·2 (130/131) | 88·0 (22/25) | 97·2 (95/97) |
| SmSEA-mock | 82·1 (46/56) | 73·3 (96/131) | 72·0 (18/25) | 30·9 (30/97) |
| SmSEA-periodate | 98·2 (55/56) | 97·7 (128/131) | 100 (25/25) | 96·9 (94/97) |

*Specificity shown in percent and in brackets (n true negatives/n) based on defined arbitrary MFI=10000 cut-off (Figure 2 C, D)
Abbreviations/sample set: Donor (no. inf) = no infection (G); PTSP = Post-Travel Screening of Parasites (B); *S. ster. = Strongyloides stercoralis* (F); STH = Soil-transmitted helminths (E)

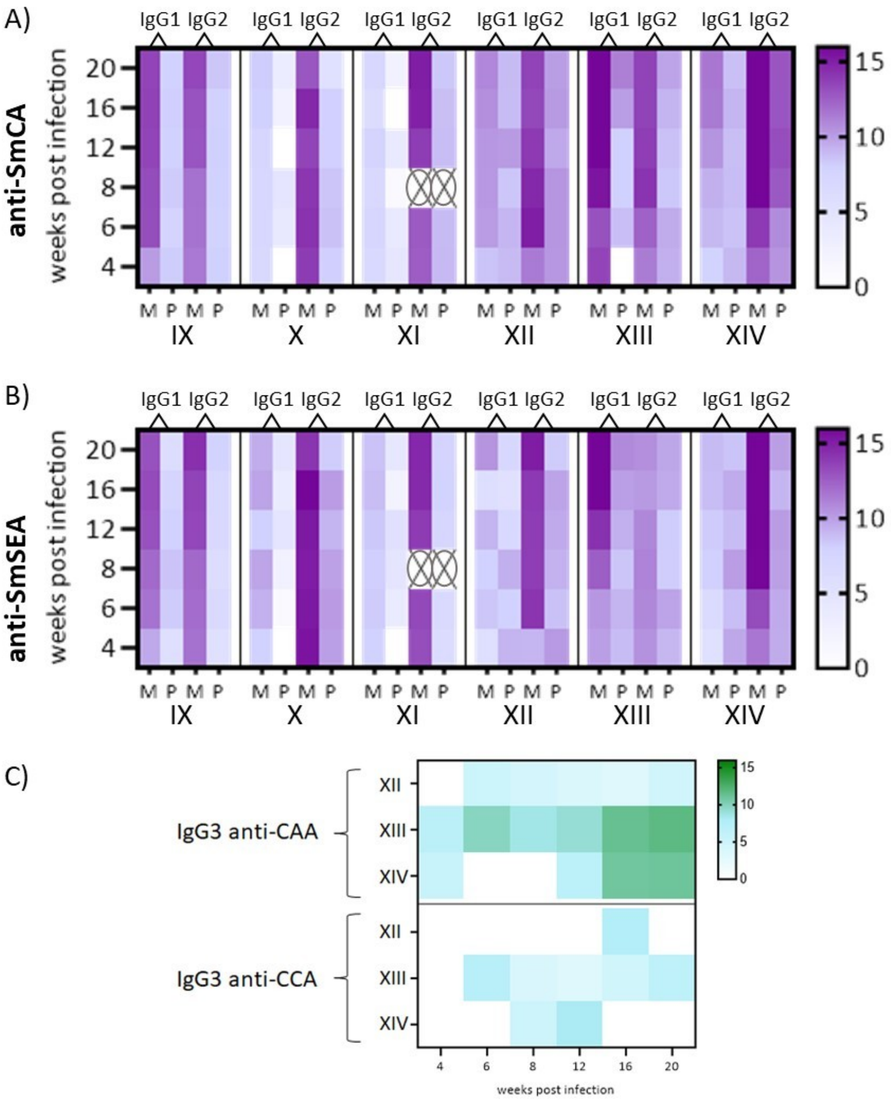


**Fig S2: IgG subclass responses in primary human schistosome infections**

Heatmaps showing levels (log2 MFI+1) of IgG1 and IgG2 specific for A) SmCA and B) SmSEA mock (M) and periodate treated (P) antigen for six individuals infected with 10 or 30 cercariae (10 cercs: IX, X, XI, 30 cercs: XII, XIII, XIV) at an early phase (weeks 4, 6, 8) and later phase (weeks 12, 16, 20) post infection. Note the highly similar pattern for SmCA and SmSEA, which is due to epitopes shared between SmCA and SmSEA (male only infection, no eggs present). No anti-SmCA/SmSEA IgG3 or IgG4 was detectable for IX, X, XI, not measured for XII, XIII, XIV. Any binding below log2 (MFI+1) = 8 is negligible. ⨂ = no data available. Comparison of periodate treated SmCA and SmSEA with their mock treated counter parts demonstrates that for both SmCA and SmSEA most antibody binding is to glycans. Periodate treatment disrupts monosaccharide ring structures with adjacent cis-hydroxyl groups, hence mediating a molecular change that abolish antibody binding to the majority of glycan epitopes for both IgG1 and IgG2 antibodies measured on microarrays. Interestingly, the IgG subclass repertoire elicited in response to infection does not change from early (week four to eight) to later stage (week 12-20) of infection but does vary on inter-individual level in terms of which subclasses are elicited (A, B). For example, individual IX has both IgG1 and IgG2 whereas X and XI predominantly induce IgG2. C) IgG3 responses in individual XII, XIII, and XIV to CAA and CCA. IgG4 was not measured. Any binding below log2 (MFI+1) = 8 is negligible.

## SECTION 3: SUPPLEMENTARY REFERENCES


1. de Boer AR, Hokke CH, Deelder AM, Wuhrer M. 2007. General microarray technique for immobilization and screening of natural glycans. Anal Chem 79:8107-13.
2. Corstjens PLAM, De Dood CJ, Kornelis D, Tjon Kon Fat EM, Wilson RA, Kariuki TM, Nyakundi RK, Loverde PT, Abrams WR, Tanke HJ, Van Lieshout L, Deelder AM, Van Dam GJ. 2014. Tools for diagnosis, monitoring and screening of *Schistosoma* infections utilizing lateral-flow based assays and upconverting phosphor labels. Parasitology 141:1841-1855.
3. de Dood CJ, Hoekstra PT, Mngara J, Kalluvya SE, van Dam GJ, Downs JA, Corstjens PLAM. 2018. Refining diagnosis of *Schistosoma haematobium* infections: antigen and antibody detection in urine. Frontiers in Immunology 9.
4. Harvey MR, Chiodo F, Noest W, Hokke CH, van der Marel GA, Codée JDC. 2021. Synthesis and antibody binding studies of *Schistosome*-derived oligo-α-(1-2)-l-fucosides. Molecules 26:2246.
5. Harvey MR. 2020. Synthesis and application of glycans unique to *S. mansoni*. PhD. Leiden University. https://hdl.handle.net/1887/138246
6. van Roon A-MM, Aguilera B, Cuenca F, van Remoortere A, van der Marel GA, Deelder AM, Overkleeft HS, Hokke CH. 2005. Synthesis and antibody-binding studies of a series of parasite fuco-oligosaccharides. Bioorganic & Medicinal Chemistry 13:3553-3564.
7. Ágoston K, Kerékgyártó J, Hajkó J, Batta G, Lefeber DJ, Kamerling JP, Vliegenthart JFG. 2002. Synthesis of fragments of the glycocalyx glycan of the parasite *Schistosoma mansoni*. Chemistry – A European Journal 8:151-161.
8. van Remoortere A, Vermeer HJ, van Roon AM, Langermans JA, Thomas AW, Wilson RA, van die I, van den Eijnden DH, Agoston K, Kerekgyarto J, Vliegenthart JF, Kamerling JP, van dam GJ, Hokke CH, Deelder AM. 2003. Dominant antibody responses to Fucalpha1-3GalNAc and Fucalpha1-2Fucalpha1-3GlcNAc containing carbohydrate epitopes in *Pan troglodytes* vaccinated and infected with *Schistosoma mansoni*. Exp Parasitol 105:219-25.
9. van Roon AM, Pannu NS, Hokke CH, Deelder AM, Abrahams JP. 2003. Crystallization and preliminary X-ray analysis of an anti-LewisX Fab fragment with and without its LewisX antigen. Acta Crystallogr D Biol Crystallogr 59:1306-9.
10. Roon A-Mv. 2005. *Schistosoma mansoni*: structural and biophysical aspects of Lewis X-antibody interactions. PhD. https://scholarlypublications.universiteitleiden.nl/handle/1887/599
11. Halkes KM, Vermeer HJ, Slaghek TM, van Hooft PA, Loof A, Kamerling JP, Vliegenthart JF. 1998. Preparation of spacer-containing di-, tri-, and tetrasaccharide fragments of the circulating anodic antigen of *Schistosoma mansoni* for diagnostic purposes. Carbohydr Res 309:175-88.
12. Vermeer HJ, Halkes KM, van Kuik JA, Kamerling JP, Vliegenthart JFG. 2000. Synthesis and conjugation of oligosaccharide fragments related to the immunologically reactive part of the circulating anodic antigen of the parasite *Schistosoma mansoni*. Journal of the Chemical Society, Perkin Transactions 1 doi:10.1039/B002083O:2249-2263.
13. van Dam GJ, Seino J, Rotmans JP, Daha MR, Deelder AM. 1993. *Schistosoma mansoni* circulating anodic antigen but not circulating cathodic antigen interacts with complement component C1q. European Journal of Immunology 23:2807-2812.
14. Janse JJ, Langenberg MCC, Kos-Van Oosterhoud J, Ozir-Fazalalikhan A, Brienen EAT, Winkel BMF, Erkens MAA, van der Beek MT, van Lieshout L, Smits HH, Webster BL, Zandvliet ML, Verbeek R, Westra IM, Meij P, Visser LG, van Diepen A, Hokke CH, Yazdanbakhsh M, Roestenberg M. 2018. Establishing the production of male *Schistosoma mansoni* cercariae for a controlled human infection model. The Journal of Infectious Diseases 218:1142-1146.
